# Correspondence

**Published:** 1904-02

**Authors:** Geo. N. Jack

**Affiliations:** Buffalo, N. Y.


### CORRESPONDENCE.

*Editor Buffalo Medical Journal:*

SIR:—Owing to the fact that some physicians of Buffalo, who are friends of mine, have stated to me that there is a feeling among some Buffalo physicians that my institution has not been conducted on a strictly ethical basis, I would like, therefore, through the medium of your journal to inform these physicians that it is the spirit and intention of this institution to be strictly ethical and regular.

I have no secrets; my methods of treating asthma are open to all physicians and they are invited to inspect the same.

GEO. N. JACK,

261 Georgia St.

Buffalo, N. Y., Jan. 12, 1904.

